# Association between metabolic score of visceral fat and carotid atherosclerosis in Chinese health screening population: a cross-sectional study

**DOI:** 10.1186/s12889-024-19186-2

**Published:** 2024-06-28

**Authors:** Jiayu Qian, Guoqing Huang, Yushan Mao

**Affiliations:** 1 Department of Endocrinology, The First Affiliated Hospital of Ningbo University, Ningbo University, Ningbo, 315000 Zhejiang China; 2 Health Science Center, Ningbo University, Ningbo, 315211 Zhejiang China

**Keywords:** Metabolic score for visceral fat, Carotid atherosclerosis, Nonlinear relationship

## Abstract

**Background:**

The metabolic score for visceral fat (METS-VF) quantifies the cumulative burden of visceral and intra-abdominal adipose tissues. However, the relationship between the METS-VF and carotid atherosclerosis (CAS) has not been extensively explored. Therefore, this study aimed to investigate the association between the METS-VF and CAS.

**Methods:**

This cross-sectional study enrolled 7089 Chinese adults who underwent physical examinations at the Zhenhai Lianhua Hospital, Zhejiang, China, in 2020. Multivariable logistic regression analysis was used to explore the linear relationship between METS-VF and CAS. Generalised additive models (GAM) were employed to evaluate potential nonlinear associations. The inflection points of METS-VF were determined using segmented logistic regression analysis optimised for maximum likelihood ratios and recursive algorithms.

**Results:**

Multivariable logistic regression analysis revealed a positive correlation between METS-VF and CAS (odds ratio [OR]: 1.824, 95% confidence interval [CI]: 1.753–1.899; *P* < 0.001). The GAM analysis confirmed a nonlinear association between them [effective degrees of freedom: 4.803, χ^2^: 876.7, *P* < 0.001], with an inflection point at a METS-VF of 8.09 (*P* < 0.001 for log-likelihood ratio test). Below this inflection point, METS-VF exhibited a significant positive association with CAS risk (OR: 1.874, 95% CI: 1.796–1.954; *P* < 0.001). Conversely, no significant association was observed when METS-VF ≥ 8.09 (OR: 0.998, 95% CI: 0.786–1.268; *P* = 0.989).

**Conclusions:**

METS-VF and CAS demonstrated a positive non-linear correlation, with the curve indicating a saturation effect at METS-VF = 8.09.

**Supplementary Information:**

The online version contains supplementary material available at 10.1186/s12889-024-19186-2.

## Background

Atherosclerosis is characterised by the thickening, hardening, and decreased elasticity of arterial walls, and is the leading cause of mortality globally, imposing substantial health burdens in both developed and developing countries [1]. Atherosclerosis is the leading cause of death worldwide, accounting for approximately 610,000 deaths annually in the United States [2]. Carotid atherosclerosis (CAS) is a manifestation of systemic atherosclerosis in the carotid arteries that predisposes to ischaemic stroke, accounting for approximately 610,000 new and 185,000 recurrent strokes annually [3]. Early identification and management of the risk factors associated with CAS can decelerate disease progression and mitigate the risk of adverse prognostic events.

Carotid ultrasonography is currently used in clinical practice for early screening and diagnosis of CAS, and the discovery of additional biomarkers associated with CAS may provide an alternative for screening and monitoring the disease [4]. Human adipose tissue is categorised into visceral adipose tissue (VAT) and subcutaneous adipose tissue (SAT) [5]. Britton et al. indicated that VAT and SAT have diametrically opposite effects on cardiovascular health, with VAT implicated in the pathogenesis of CAS [6, 7]. The metabolic score for visceral fat (METS-VF) is an index for the quantification of VAT, which is designed through complex nonlinear modelling that considers the metabolic score for insulin resistance (METS-IR), waist-to-height ratio (WHtR), age, and sex, and uses dual X-ray absorptiometry as a reference standard [8]. Moreover, this innovative index has been corroborated through validation with magnetic resonance imaging (MRI) and bioelectrical impedance analysis [8]. Neftali et al., reported that METS-VF identifies individuals with high VAT (VAT > 127 cm^2^ for women, > 152.7 cm^2^ for men), with an optimal area under the curve (AUC) (0.84, 95% CI: 0.81–0.87, *P* < 0.001) [9].

METS-VF is closely associated with metabolic diseases, and is a reliable predictor for the development of type 2 diabetes (AUC: 0.690, 95% CI: 0.682–0.698), metabolic syndrome (AUC: 0.952, 95% CI: 0.951–0.953), non-alcoholic fatty liver disease (NAFLD) (AUC: 0.812, 95% CI: 0.802–0.823), and chronic kidney disease (AUC: 0.634, 95% CI: 0.589–0.680) [10–13]. Moreover, METS-VF exhibits a robust association with several diseases, including kidney stones, asthma, hypertension, and hyperuricaemia [14–17].

Existing studies have mainly focused on the linear relationship between the METS-VF and diseases. However, real-world data on the relationships between independent variables and outcome events often deviate from simple linear correlations and potentially follow more intricate curvilinear patterns [18]. Few studies have reported an association between METS-VF and CAS. This study aimed to investigate the exact relationship between METS-VF and CAS.

## Materials and methods

### Study population

**Fig. 1:**
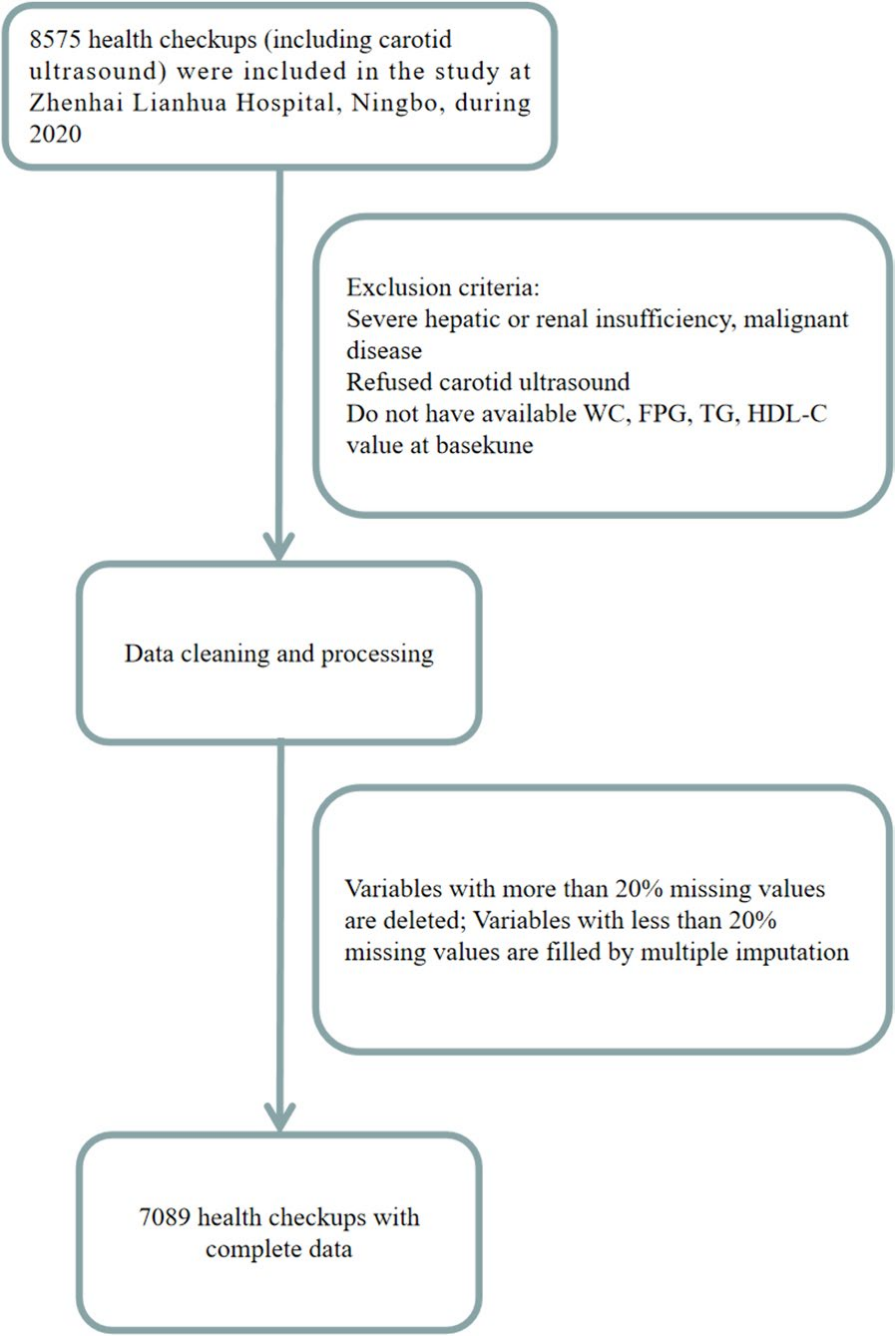
Flow diagram of the study

This study initially included 8,575 adults (age: 21–98 years) who had participated in health checkups, which incorporated carotid ultrasound examinations, at Zhenhai Lianhua Hospital, Ningbo, China, in 2020. The participants’ data were collected using the hospital’s electronic medical record system. Individuals with hepatic or renal dysfunction; malignancies; those who were unwilling to participate in carotid ultrasound examination; and those with missing baseline measurements of waist circumference (WC), fasting blood glucose (FBG), triglycerides (TG), and high-density lipoprotein cholesterol (HDL-C), were excluded. Variables with more than 20% missing data were removed, and multiple interpolations were applied to the remaining incomplete data (Additional Fig. A1). Finally, 7,089 participants were included in the study. A comprehensive illustration of the methodology used in this study is shown in Fig. 1.

### Definition

CAS is defined as an increase in carotid intima-media thickness of ≥ 1 mm or plaque formation [19]. CAS was detected using carotid ultrasonography, and the findings were corroborated by two independent sonographers. Dyslipidaemia was defined as total cholesterol (TC) ≥ 5.17 mmol/L, TG ≥ 1.7 mmol/L, or LDL ≥ 3.37 mmol/L [20].

Body mass index (BMI) was calculated as:

$$\text{BMI}=\text{weight }(\text{kg})/{\text{height}}^{2} ({\text{m}}^{2})$$

WhtR was calculated as [21]:

$$\text{WhtR}=\text{WC }(\text{cm})/\text{height }(\text{cm})$$

The METS-IR was calculated using the expression [22]:

$$\text{METS}-\text{IR}=(\text{Ln}((2\times \text{FBG})+\text{TG})\times \text{BMI})/(\text{Ln}(\text{HDL}-\text{C}))$$

Units of biochemical data were converted to mg/dL.

The calculation of METS-VF adheres to the specified equation [8]:

$$\text{METS}-\text{VF}=4.466+0.011\times \left[({\text{Ln}(\text{METS}-\text{IR}))}^{3}\right]+3.239\times [{(\text{Ln}(\text{WHtr}))}^{3}] +0.319\times (\text{Sex})+0.594\times (\text{Ln}(\text{Age}))$$

In this model, the variable ‘sex’ was encoded as a binary indicator (1 and 0 for men and women, respectively). An example calculation is provided in Additional file 2.

### Clinical baseline data

Baseline clinical data collection and evaluation were performed using established protocols from previous studies [23]. Baseline clinical data for this study were determined based on previous research [24]. Personal demographic information, including name, sex, age, and medical history, was collected using structured questionnaires. Height and weight were measured using the HGM-700 (Zhengzhou Shengyuan, China), and participants were asked to remove shoes and heavy clothing for weight assessment and to stand with their feet together and heels, buttocks, shoulders, and head against the measuring instrument for height assessment. WC was assessed by positioning the measuring tape midway between the lowest rib and the iliac crest, with the participants in a standing position and relaxed breathing. Blood pressure was determined using an electronic sphygmomanometer (Omron Corp., Kyoto, Japan) after the participants were allowed to rest for 5–10 min with their arms positioned at heart level on a horizontal surface. Following an 8–12 h overnight fast, venous blood samples were collected by professional nurses for analysis. Routine biochemical parameters were assessed using an AU640 analyser (Olympus).

### Statistical analysis

The normality of the data was analysed using the Kolmogorov–Smirnov test. Normally and non-normally distributed continuous variables were presented as the mean (standard deviation) and median (interquartile range), respectively. Categorical variables were expressed as frequencies (percentages). Clinical baseline characteristics of the study population were stratified by quartiles of METS-VF as follows: Q1 ≤ 7.47; 7.47 < Q2 ≤ 7.70; 7.70 < Q3 ≤ 7.88; and Q4 > 7.88. Group balance was assessed using standardised mean differences (SMD). Univariate analyses were conducted using independent sample *t*-tests for normally distributed continuous variables, Mann–Whitney *U*-tests for non-normally distributed continuous variables, and Chi-squared tests for categorical variables. All statistical tests were two-tailed, and a *P* < 0.05 indicated statistical significance. All statistical analyses were performed using the R software, version 4.2.3 (http://www.R-project.org/; R Core Team, Vienna, Austria).

Multivariable logistic regression analysis was used to explore the linear relationship between METS-VF and CAS. Covariates associated with CAS were identified using the change-in-estimate method for the odds ratio (OR) [25], specifically when the introduction or removal of a variable resulted in a change in the OR (METS-VF) exceeding 10% (Additional Fig. S2). Furthermore, WHtr, sex, age, BMI, HDL-C, FBG, TG, and METS-IR were used in the computation of METS-VF; therefore, these variables were not incorporated as covariates in the model.

Sensitivity analysis was performed to enhance the reliability of the findings. METS-VF was converted into a categorical variable based on quartiles, and trend tests were used to examine the pattern of change in the effect estimates (OR) of METS-VF (categorical variable). Given that age and obesity are well-known risk factors for CAS [26], further sensitivity analyses were performed to exclude individuals aged ≥ 60 years with a BMI ≥ 24 kg/m^2^. Additionally, E-values were computed to assess the potential impact of unmeasured confounding factors on study outcomes.

A generalised additive model (GAM) and smooth curve fitting were employed to explore the nonlinear relationship between METS-VF and CAS. Segmented logistic regression with log-likelihood ratio tests and recursive algorithms was applied to ascertain the threshold (inflection point) for METS-VF. Subsequently, subgroup analyses were conducted to explore the robustness of the findings and the interactions between METS-VF and various variables.

## Results

### Baseline characteristics of participants

This study comprised 7089 participants (4717 men and 2372 women), including 2094 individuals with CAS. Table 1 summarises the basic information, laboratory data, and CAS prevalence. The prevalence of CAS, proportion of men, BMI, WC, FBG, systolic blood pressure (SBP), TG, homocysteine, METS-IR, WHtr, aspartate aminotransferase, globulin, serum creatinine (SCR), serum uric acid, total bilirubin, direct bilirubin, total bile acids, and blood urea nitrogen were higher in the Q4 group than in the other three groups. In contrast, the Q1 group had higher HDL-C and apolipoprotein levels than the other three groups.

**Table 1:** The baseline characteristics of participants

METS-VF	Overall	Q1(≤ 7.47)	Q2(7.47 to ≤ 7.70)	Q3(7.40 to ≤ 7.88)	Q4(> 7.88)	SMD
*N*	7089	1779	1863	1734	1713	
Male, n (%)	4717 (66.5)	566 (31.9)	1126 (60.4)	1458 (84.1)	1567 (91.5)	0.825
Age, years	53.00 (42.00, 66.00)	36.00 (30.00, 50.00)	48.00 (38.00, 58.50)	55.00 (50.00, 64.00)	70.00 (64.00, 79.00)	1.570
WC, cm	80.80 (9.57)	72.39 (7.49)	79.84 (7.98)	83.66 (7.62)	87.69 (7.88)	1.074
BMI, kg/m^2^	23.42 (3.07)	21.57 (2.54)	23.37 (2.95)	24.07 (2.86)	24.76 (2.95)	0.616
SBP, mmHg	129.61 (17.07)	118.51 (13.29)	127.33 (14.77)	132.49 (15.61)	140.71 (16.54)	0.796
DBP, mmHg	77.82 (10.88)	72.14 (9.38)	77.54 (10.05)	81.39 (10.56)	80.42 (11.08)	0.504
FBG, mmol/L	5.38 (5.07, 5.79)	5.12 (4.89, 5.37)	5.32 (5.05, 5.65)	5.47 (5.17, 5.89)	5.74 (5.36, 6.41)	0.689
TC, mmol/L	5.20 (1.03)	5.05 (0.96)	5.42 (1.01)	5.34 (1.03)	4.98 (1.06)	0.264
TG, mmol/L	1.27 (0.91, 1.79)	0.92 (0.72, 1.24)	1.33 (0.96, 1.76)	1.45 (1.06, 2.07)	1.47 (1.04, 2.10)	0.535
HDL-C, mmol/L	1.21 (1.02, 1.47)	1.44 (1.22, 1.70)	1.23 (1.06, 1.47)	1.15 (0.99, 1.37)	1.06 (0.90, 1.26)	0.589
LDL-C, mmol/L	3.00 (0.82)	2.80 (0.73)	3.18 (0.79)	3.12 (0.83)	2.87 (0.85)	0.289
Apo-A1,mmol/L	1.42 (1.28, 1.57)	1.48 (1.35, 1.64)	1.43 (1.32, 1.59)	1.41 (1.27, 1.56)	1.33 (1.20, 1.46)	0.381
Apo-B, mmol/L	0.96 (0.78, 1.15)	0.84 (0.70, 1.00)	1.00 (0.85, 1.18)	1.03 (0.85, 1.20)	0.97 (0.78, 1.16)	0.380
HCY, mmol/L	12.90 (11.30, 14.80)	11.40 (10.00, 13.20)	12.40 (11.20, 14.10)	13.20 (11.83, 14.90)	14.30 (12.70, 16.30)	0.530
METS-IR	43.74 (37.65, 50.04)	36.65 (32.90, 41.37)	43.07 (38.32, 48.48)	46.29 (40.90, 51.85)	49.12 (43.94, 55.29)	0.848
WHtr	1.24 (1.16, 1.32)	1.24 (1.16, 1.31)	1.22 (1.14, 1.32)	1.22 (1.15, 1.30)	1.27 (1.20, 1.37)	0.229
ALT, IU/L	20.00 (14.00, 28.00)	15.00 (12.00, 21.00)	21.00 (15.00, 29.00)	22.00 (16.00, 32.00)	21.00 (16.00, 30.00)	0.352
AST, IU/L	22.00 (19.00, 27.00)	20.00 (17.00, 23.00)	23.00 (19.00, 27.00)	23.00 (20.00, 28.00)	24.00 (21.00, 29.00)	0.398
TP, g/L	74.12 (3.88)	74.12 (3.60)	74.53 (3.88)	73.90 (3.86)	73.88 (4.15)	0.094
ALB, g/L	45.11 (2.20)	45.43 (2.18)	45.55 (2.04)	45.13 (2.05)	44.26 (2.28)	0.322
GLB, g/L	29.01 (3.43)	28.69 (3.09)	28.98 (3.51)	28.76 (3.42)	29.62 (3.63)	0.144
GGT, IU/L	23.00 (17.00, 34.00)	17.00 (13.00, 22.00)	23.00 (17.00, 33.00)	27.00 (20.00, 41.00)	26.00 (19.00, 40.00)	0.502
SCR, μmol/L	70.00 (61.00, 79.00)	61.00 (54.00, 71.00)	68.00 (60.00, 76.00)	73.00 (65.00, 81.00)	76.00 (68.00, 86.00)	0.613
SUA, μmol/L	353.27 (83.20)	304.40 (71.41)	353.17 (76.87)	373.04 (78.04)	384.17 (83.30)	0.564
TBil, μmol/L	13.50 (10.50, 17.40)	12.30 (9.60, 16.10)	13.40 (10.40, 17.00)	14.00 (11.00, 17.80)	14.30 (11.10, 18.60)	0.179
DBil, μmol/L	3.00 (2.30, 4.00)	2.80 (2.20, 3.80)	2.90 (2.30, 3.70)	3.00 (2.40, 3.90)	3.30 (2.60, 4.40)	0.194
IBil, μmol/L	10.40 (7.90, 13.60)	9.50 (7.30, 12.50)	10.50 (8.00, 13.40)	10.80 (8.40, 14.20)	10.80 (8.30, 14.20)	0.168
TBA, μmol/L	2.71 (1.86, 4.09)	2.47 (1.71, 3.81)	2.50 (1.76, 3.77)	2.78 (1.90, 4.12)	3.18 (2.18, 4.74)	0.215
BUN, mmol/L	4.95 (4.22, 5.83)	4.52 (3.88, 5.30)	4.92 (4.24, 5.72)	5.05 (4.35, 5.87)	5.41 (4.56, 6.48)	0.401
CAS, n(%)	2094 (29.5)	41 ( 2.3)	244 (13.1)	595 (34.3)	1214 (70.9)	1.016

*WC* Waist circumference, *BMI* Body mass index, *SBP* Systolic blood pressure, *DBP* Diastolic blood pressure, *FBG* Fasting blood glucose, *TC* Total cholesterol, *TG* Triglycerides, *HDL-C* High-density lipoprotein cholesterol, *LDL-C* Low-density lipoprotein cholesterol, *Apo-A1* Apolipoprotein-A1, *Apo-B* Apolipoprotein-B, *HCY* Homocysteine, *METS-IR* Metabolic score for insulin resistance, *WHtr* Waist-to-height ratio, *ALT* Alanine aminotransferase, *AST* Aspartate aminotransferase, *TP* Total protein, *ALB* Albumin, *GLB* Globulin, *GGT* Gamma-glutamyl transpeptidase, *SCR* Serum creatinine, *SUA* Seru uric acid, *TBil* Total bilirubin, *DBil* Direct bilirubin, *IBil* Indirect bilirubin, *TBA* Total bile acids, *BUN* Blood urea nitrogen

### Prevalence of CAS

**Fig. 2:**
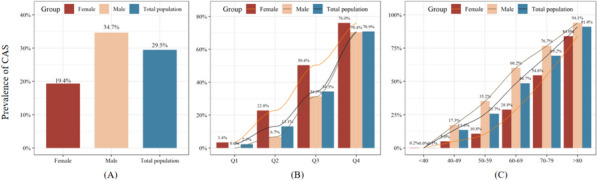
Prevalence of CAS in different populations. **A** Prevalence of CAS among different gender groups; **B** Prevalence of CAS across quartiles of METS-VF; **C** Prevalence of CAS among various age groups

As shown in Fig. 2A, 29.5% of the participants had CAS, with a higher prevalence in men (34.7%) than in women (19.4%). Notably, there was an increasing trend in the prevalence of CAS from Q1 to Q4 (*P* for trend < 0.001) (Fig. 2B). Furthermore, the disparity in the prevalence rates among the quartiles was substantial, with an SMD of 1.016 (Table 1) [27]. Figure 2C shows the age-related increase in CAS prevalence, with a significant trend (*P* for trend < 0.001) and a clear sex disparity within identical age cohorts.

### Univariate analysis of CAS

Univariate analysis revealed that the median age, SBP, and METS-VF of CAS patients were significantly higher than those of the non-CAS population, as shown in Table 2 (*P* < 0.001, SMD > 0.65) [27]. No significant differences in low-density lipoprotein cholesterol (LDL-C) were observed between the two participant groups (*P* > 0.05). Furthermore, groups with and without CAS exhibited commendable balance with respect to LDL-C, alanine aminotransferase, total protein, and indirect bilirubin, with negligible differences between the groups (SMD < 0.1).

**Table 2:** Univariate analysis of carotid atherosclerosis

Variables	Normal	CAS	*P*-value	SMD
*N*	4995	2094		
Male, n(%)	3082 (61.7)	1635 (78.1)	< 0.001	0.363
Age, years	49.00 (36.00, 57.00)	68.00 (58.00, 77.75)	< 0.001	1.553
WC, cm	79.23 (9.54)	84.54 (8.55)	< 0.001	0.587
BMI, kg/m^2^	23.13 (3.10)	24.13 (2.87)	< 0.001	0.335
SBP, mmHg	125.79 (15.54)	138.71 (17.12)	< 0.001	0.790
DBP, mmHg	76.99 (10.75)	79.79 (10.96)	< 0.001	0.258
FBG, mmol/L	5.29 (5.02, 5.64)	5.62 (5.27, 6.20)	< 0.001	0.591
TC, mmol/L	5.23 (0.99)	5.12 (1.12)	< 0.001	0.103
TG, mmol/L	1.21 (0.87, 1.71)	1.40 (1.01, 1.95)	< 0.001	0.247
HDL-C, mmol/L	1.25 (1.06, 1.51)	1.12 (0.96, 1.34)	< 0.001	0.354
LDL-C, mmol/L	3.01 (0.78)	2.97 (0.90)	0.067	0.046
Apo-A1, mmol/L	1.44 (1.31, 1.59)	1.36 (1.23, 1.50)	< 0.001	0.283
Apo-B, mmol/L	0.95 (0.78, 1.13)	0.99 (0.79, 1.19)	< 0.001	0.118
HCY, mmol/L	12.40 (11.00, 14.20)	13.90 (12.30, 15.90)	< 0.001	0.515
METS-IR	42.25 (36.30, 48.54)	46.97 (41.20, 52.80)	< 0.001	0.462
WHtr	1.22 (1.15, 1.31)	1.27 (1.19, 1.36)	< 0.001	0.367
METS-VF	7.58 (7.40, 7.76)	7.92 (7.79, 8.04)	< 0.001	1.512
ALT, IU/L	19.00 (14.00, 28.00)	20.00 (15.00, 29.00)	< 0.001	0.081
AST, IU/L	22.00 (19.00, 26.00)	24.00 (20.00, 29.00)	< 0.001	0.290
TP, g/L	74.22 (3.79)	73.87 (4.09)	0.001	0.087
ALB, g/L	45.40 (44.10, 46.80)	44.50 (43.00, 46.00)	< 0.001	0.482
GLB, g/L	28.80 (3.33)	29.50 (3.62)	< 0.001	0.202
GGT, IU/L	22.00 (16.00, 32.00)	25.00 (19.00, 38.00)	< 0.001	0.235
SCR, μmol/L	68.35 (12.80)	76.36 (15.25)	< 0.001	0.569
SUA, μmol/L	334.00 (287.00, 390.00)	377.00 (322.25, 433.00)	< 0.001	0.483
TBil, μmol/L	13.30 (10.40, 17.20)	13.80 (10.80, 18.00)	< 0.001	0.106
DBil, μmol/L	3.00 (2.30, 3.90)	3.10 (2.50, 4.10)	< 0.001	0.172
IBil, μmol/L	10.30 (7.90, 13.50)	10.60 (8.10, 13.90)	0.007	0.076
TBA, μmol/L	2.60 (1.82, 3.93)	3.00 (2.01, 4.55)	< 0.001	0.220
BUN, mmol/L	4.82 (4.13, 5.62)	5.34 (4.52, 6.40)	< 0.001	0.452

*WC* Waist circumference, *BMI* Body mass index, *SBP* Systolic blood pressure, *DBP* Diastolic blood pressure, *FBG* Fasting blood glucose, *TC* Total cholesterol, *TG* Triglycerides, *HDL-C* High-density lipoprotein cholesterol, *LDL-C* Low-density lipoprotein cholesterol, *Apo-A1* Apolipoprotein-A1, *Apo-B* Apolipoprotein-B, *HCY* Homocysteine, *METS-IR* Metabolic score for insulin resistance, *WHtr* Waist-to-height ratio, *METS-VF* Metabolic score for visceral fat, *ALT* Alanine aminotransferase, *AST* Aspartate aminotransferase, *TP* Total protein, *ALB* Albumin, *GLB* Globulin, *GGT* Gamma-glutamyl transpeptidase, *SCR* Serum creatinine, *SUA* Seru uric acid, *TBil* Total bilirubin, *DBil* Direct bilirubin, *IBil* Indirect bilirubin, *TBA* Total bile acids, *BUN* Blood urea nitrogen

### Association between METS-VF and CAS

The multivariable logistic regression model with the OR and 95% confidence interval (CI) for the association between METS-VF and CAS is presented in Table 3. After screening the variables using the change-in-estimate method, the final variables included in the multivariable logistic regression model were SBP, albumin (ALB), SCR, gamma glutamyl transferase (GGT), apolipoprotein-B (Apo-B), and diastolic blood pressure (DBP), with OR changes of -35.6%, -24.6%, -23.6%, 10.2%, -12.3%, and -11.4%, respectively (Additional Fig. A2). After adjusting for these variables, the OR (95% CI) for CAS associated with METS-VF (per0.1) was 1.824 (1.753–1.899), which indicated that every 0.1 unit increase in METS-VF increased the risk of CAS by 82.4%.

**Table 3:** The multivariable logistic regression analysis of METS-VF and carotid atherosclerosis

Variables	OR (95%CI)	*P*-value
SBP, mmHg	1.033 (1.027 – 1.039)	< 0.001
DBP, mmHg	0.967 (0.959 – 0.975)	< 0.001
ALB, g/L	0.895 (0.867 – 0.925)	< 0.001
GGT, IU/L	0.996 (0.991 – 0.999)	0.031
SCR, μmol/L	1.016 (1.011 – 1.021)	< 0.001
Apo-B, mmol/L	1.432 (1.107 – 1.851)	0.006
METS-VF (per 0.1)	1.824 (1.753 – 1.899)	< 0.001

*Apo-B* Apolipoprotein-B, *SCR* Serum creatinine, *SBP* Systolic blood pressure, *ALB* Albumin, *GGT* Gamma-glutamyl transpeptidase, *DBP* Diastolic blood pressure, *METS-VF* Metabolic score for visceral fat, *OR* Odds ratio, *CI* Confidence

### Sensitivity analysis

After converting the METS-VF from a continuous variable to a categorical variable, it was reintroduced into the model. The results revealed that the *P* for the trend was not equal when METS-VF was converted to a categorical variable, suggesting that the association between METS-VF and CAS risk may be nonlinear (Table 4). Collecting all the confounding variables during the actual data collection remained challenging. The E-value was computed to evaluate the influence of the unobserved confounders on the study findings. The resulting E-value (3.050) indicated that unmeasured confounding factors would have needed to be associated with both METS-VF and the CAS with an OR of at least 3.05 to fully explain the observed OR of 1.824. This demonstrates that even in the presence of unmeasured confounders, the results remained relatively reliable.

**Table 4:** Relationship between METS-VF and CAS in different sensitivity analyses

Variables	Model I		Model II	
	**OR (95%CI)**	***P*-value**	**OR (95%CI)**	***P*-value**
METS-VF (per0.1)	1.773 (1.659 – 1.899)	< 0.001	1.771 (1.684 – 1.865)	< 0.001
METS-VF (Quintile)				
Q1	ref		ref	
Q2	2.357 (1.402 – 4.131)	0.002	4.702 (2.871 – 8.135)	< 0.001
Q3	6.101 (3.765 – 10.42)	< 0.001	13.234 (8.255 – 22.549)	< 0.001
Q4	17.275 (10.697 – 29.448)	< 0.001	47.821 (29.727 – 81.742)	< 0.001
*P* for trend	2.678 (2.365 – 3.042)	< 0.001	3.388 (3.034 – 3.794)	< 0.001

Model I was sensitivity analysis in participants with BMI < 24 kg/m^2^. We adjusted Apo-B, SCR, SBP, ALB, GGT, and DBP

Model II was sensitivity analysis in participants aged < 60 years. We adjusted Apo-B, SCR, SBP, ALB, GGT, and DBP

*OR* Odds ratio, *CI* Confidence, *ref* Reference, *METS-VF* Metabolic score for visceral fat

Furthermore, sensitivity analysis for individuals with a BMI < 24 kg/m^2^ (Model I), after adjusting for potential confounders, revealed a significant positive association between METS-VF and CAS risk (OR: 1.733, 95% CI: 1.659–1.899; *P* < 0.001). Similarly, sensitivity analysis for individuals aged ≤ 60 years (Model II) also indicated a positive correlation between METS-VF and NAFLD risk (OR: 1.771, 95% CI: 1.684–1.865; *P* < 0.001).

### Nonlinear relationship between METS-VF and CAS

**Fig. 3:**
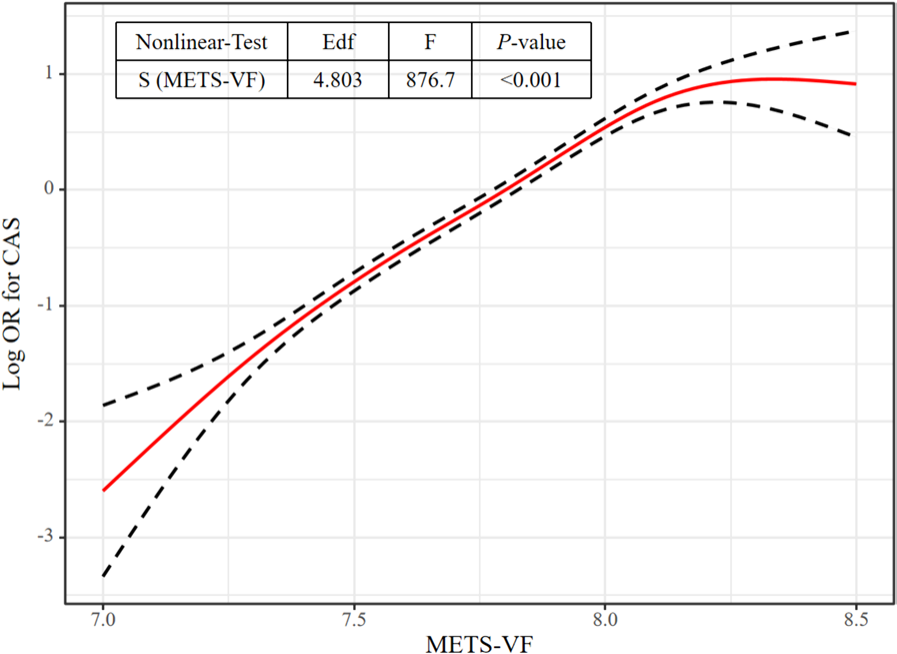
The nonlinear relationship between METS-VF and CAS. A nonlinear relationship between them was detected after adjusting for Apo-B, SCR, SBP, ALB, GGT, and DBP

**Fig. 4:**
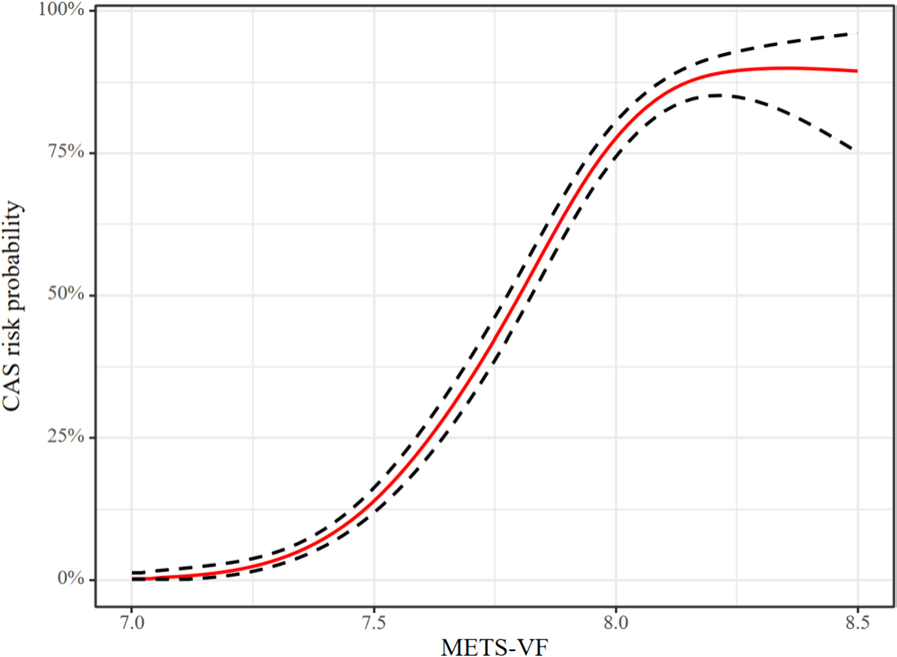
The nonlinear relationship between METS-VF and CAS risk probability. A nonlinear relationship between them was detected after adjusting for Apo-B, SCR, SBP, ALB, GGT, and DBP

The outcomes of multivariable logistic regression analysis using GAM and smooth curve fitting to investigate the potential nonlinear relationship between METS-VF and CAS are shown in Figs. 3 and 4. Figure 3 shows the Log OR for CAS on the vertical axis, whereas Fig. 4 shows the CAS risk probability. After adjusting for confounders, the curve in Fig. 3 indicates a nonlinear relationship between METS-VF and CAS risk, with an estimated degree of freedom of 4.803. The two-piece logistic regression model found an inflection point of 8.09 for METS-VF (*P* for log-likelihood ratio test < 0.001). The correlation between METS-VF and CAS was markedly positive when the METS-VF was < 8.09 (OR: 1.874, 95% CI: 1.796–1.954; *P* < 0.001). Conversely, for METS-VF ≥ 8.09, the relationship was determined to be statistically non-significant (OR: 0.998, 95% CI: 0.786–1.268; *P* = 0.989) (Table 5).

**Table 5:** Threshold effect analysis METS-VF and carotid atherosclerosis

	**OR (95%CI)**	***P*-value**
Fitting model by multivariable logistic regression (per 0.1)	1.824 (1.753 – 1.899)	< 0.001
Fitting model by two-piece multivariable logistic regression (per 0.1)		
Inflection points of METS-VF	8.09	
< 8.09	1.874 (1.796 – 1.954)	< 0.001
≥ 8.09	0.998 (0.786 – 1.268)	0.989
Difference between effects on either side of the inflection point	0.533 (0.414 – 0.685)	< 0.001
P for log-likelihood ratio test		< 0.001

We adjusted Apo-B, SCR, SBP, ALB, GGT, and DBP

*OR* Odds ratio, *CI* Confidence, *METS-VF* Metabolic score for visceral fat

### Subgroup analysis

A subgroup analysis was performed to identify additional risk factors that might affect the association between METS-VF and CAS. Sex, age, BMI, SBP, DBP, FBG level, and blood lipid status were selected as stratification factors. Dyslipidaemia was defined as having TC ≥ 5.17 mmol/L, TG ≥ 1.7 mmol/L, or LDL ≥ 3.37 mmol/L [20]. The interactions of these factors with METS-VF for CAS risk were explored (Table 6). This analysis indicated that DBP did not modify the association between METS-VF and CAS risk. In contrast, sex, age, BMI, SBP, FBG, and blood lipid levels affected the association between METS-VF and CAS risk. Furthermore, the relationship between METS-VF and CAS was more pronounced in individuals aged < 60 years, men, and those with a SBP < 140 mmHg, a FBG < 7 mmol/L, a BMI ≥ 24 kg/m^2^, and a normal lipid profile.

**Table 6:** Effect size of METS-VF on CAS in different subgroups

Characteristic	N	OR (95%CI)	*P*-value	*P* for interaction
Gender				
Male	4717	2.208 (2.081 – 2.346)	< 0.001	< 0.001
Female	2372	1.734 (1.612 – 1.872)	< 0.001	
Age, years				
< 60	4608	1.773 (1.659 – 1.899)	< 0.001	< 0.001
≥ 60	2481	1.477 (1.392 – 1.569)	< 0.001	
BMI, kg/m^2^				
< 24	4252	1.771 (1.684 – 1.865)	< 0.001	0.004
≥ 24	2837	2.008 (1.877 – 2.153)	< 0.001	
SBP, mmHg				
< 140	5258	1.925 (1.835 – 2.023)	< 0.001	0.015
≥ 140	1831	1.715 (1.603 – 1.840)	< 0.001	
DBP, mmHg				
< 90	6083	1.840 (1.764 – 1.923)	< 0.001	0.684
≥ 90	1006	1.792 (1.614 – 2.001)	< 0.001	
FBG, mmol/L				
<7	6762	1.830( 1.756 – 1.908)	< 0.001	0.040
≥ 7	327	1.558( 1.320 – 1.861)	< 0.001	
Blood lipid status				
Normal	2897	1.937( 1.815 – 2.072)	< 0.001	0.007
Dyslipidemia	4192	1.761( 1.675 – 1.855)	< 0.001	

Note 1: Above model adjusted for Apo-B, SCR, SBP, ALB, GGT, and DBP

Note 2: In each case, the model is not adjusted for the stratification variable

Note 3: Dyslipidemia is defined as TC ≥ 5.17 mmol/L or TG ≥ 1.7 mmol/L or LDL ≥ 3.37 mmol/L

## Discussion

This study included 7,089 individuals who had participated in health screening at Zhenhai Lianhua Hospital, Zhejiang, China, in 2020. The prevalence of CAS in the overall population was 29.5%, with a higher incidence in men than in women (34.7% vs. 19.4%; *P* < 0.001). Additionally, the prevalence of CAS demonstrated an upward trend with increasing age and METS-VF quartile (P for trend < 0.001). After adjusting for confounding factors, multivariable logistic regression analysis indicated that each increment of 0.1 in METS-VF corresponded to an 82.4% increase in CAS risk. Furthermore, GAM revealed a nonlinear relationship between METS-VF and the risk of CAS, with a change point identified (8.09) through segmented logistic regression with log-likelihood ratio tests. When the METS-VF was < 8.09, it was positively associated with the risk of CAS (OR: 1.874, 95% CI: 1.796–1.954; *P* < 0.001). Subgroup analysis revealed significant interactions between the METS-VF and variables such as sex, age, BMI, SBP, FBG, and lipid levels (*P* < 0.05).

Active prevention and management of the factors associated with CAS can significantly mitigate the risk of this condition. Multivariate logistic regression analysis revealed that Apo-B, SBP, DBP, SCR, ALB, GGT and METS-VF levels were strongly associated with CAS risk. SBP has been widely recognised as a risk factor for CAS [28, 29]. Early detection and proactive intervention for hypertension can effectively prevent the occurrence of CAS and contribute to an improved long-term prognosis. Our findings indicated that every 10 mmHg increase in SBP increased the risk of CAS by 33%. A causal relationship between Apo-B and the risk of atherosclerotic cardiovascular disease has been reported [30], which is consistent with our results. Moreover, reduced renal function is a risk indicator for the onset of peripheral arterial disease [31], and our analysis showed a positive correlation between SCR levels and the risk of developing CAS (OR: 1.016, 95% CI: 1.011–1.021; *P* < 0.001).

VAT is more metabolically active than SAT and is more likely to increase the risk of atherosclerosis as a proinflammatory tissue [32]. MRI remains the gold standard for accurately assessing VAT; however, its high cost and impracticality limit its broad applications. Although various alternative indices for VAT based on simple anthropometric measurements, such as BMI, WHtR, WC, and waist-to-hip ratio, have been developed, these indices only provide a rough estimate of VAT content from the perspective of body fat distribution and do not adequately reflect the impact of VAT on metabolism [33]. METS-VF is a clinical estimator of VAT and is more accurate than other traditional visceral fat indices in estimating VAT [8, 9]. Peng Yu et al. showed that METS-VF was associated with a risk of chronic kidney disease (OR: 2.102, 95% CI: 1.653–2.674, *P* < 0.001) [12]. Moreover, METS-VF is significantly associated with the risk of hyperuricaemia in non-obese individuals, with an OR of 1.777 (95% CI: 1.318–2.396) in women and 1.228 (95% CI: 1.037–1.454) in men [17]. Our findings elucidated a positive correlation between METS-VF and CAS risk (OR: 1.016, 95% CI: 1.011–1.021; *P* < 0.001). Recent studies have revealed a nonlinear relationship between METS-VF and type 2 diabetes and erectile dysfunction [11, 34]. Similarly, our study identified a nonlinear association between METS-VF and CAS.

The METS-VF offers potential advantages for assessing CAS. First, advanced age is a well-known risk factor for CAS [35], and our findings demonstrated a significant increase in the prevalence of CAS with advancing age. Because the METS-VF incorporates the impact of age, it provides a more comprehensive assessment of CAS risk than other simple anthropometric indices (such as BMI, WHtR, WC, and waist-to-hip ratio). Second, METS-VF is a strong predictor of type 2 diabetes [11]. Notably, the METS-IR demonstrated efficacy in reflecting the severity of insulin resistance and was advantageous for evaluating adverse outcomes in patients with type 2 diabetes [22]. The main cause of type 2 diabetes is insulin resistance, which in turn contributes to atherosclerosis [36, 37]. Furthermore, the mechanism underlying the association between METS-VF and CAS was hypothesised. METS-VF can accurately quantify VAT [8]. Long-term deposition of adipose tissue leads to the excretion of large amounts of non-esterified fatty acids, glycerol, hormones, and proinflammatory cytokines, which induce oxidative stress and endoplasmic reticulum stress, resulting in adipocyte loss, metabolic disturbances, and ultimately insulin resistance [38]. Insulin resistance is a known risk factor for CAS [36, 37], and elevated fasting serum insulin levels are associated with intraplaque haemorrhage in carotid atherosclerotic lesions [39–41]. In addition, dipose tissue-associated inflammatory factors play an important role in the pathogenesis of CAS. Elevated circulating levels of resistin (proinflammatory adipokine) is an important biomarker of CAS pathogenesis [42, 43]. As part of the VAT, perivascular adipose tissue (PVAT) engages in bidirectional crosstalk with the vascular wall [44]. Atherosclerotic plaques or inflamed vascular walls can lead to adipocyte dedifferentiation and reduced lipid storage in the PVAT, and these changes may exacerbate perivascular inflammation [45].

Despite these notable findings, our study had certain limitations. First, the cross-sectional study design precludes definitive conclusions regarding the causality or directional nature of the observed associations. Furthermore, owing to the absence of data on the participants’ medical histories and medication use, this study did not account for the potential impact of preexisting conditions and pharmacological interventions on the outcomes. Despite this caveat, the calculated E-values suggest that the probability of unmeasured confounders driving the outcomes is improbable.

## Conclusions

After adjusting for confounding factors, a nonlinear relationship was observed between METS-VF and CAS risk, with a METS-VF risk threshold of 8.09. Moreover, this correlation was notably stronger among men aged < 60 years with SBP < 140 mmHg, FBG < 7 mmol/L, BMI ≥ 24 kg/m^2^, and normal lipid levels.

### Supplementary Information

Supplementary Material 1.

Supplementary Material 2.

## Data Availability

The relevant data are available from the corresponding author upon reasonable request.

## Abbreviations

METS-VF: Metabolic score for visceral fat METS-IR: metabolic score for insulin resistance
WHtr: Waist-to-height ratio
CAS: Carotid atherosclerosis
FBG: Fasting blood glucose
SBP: Systolic blood pressure
DBP: Diastolic blood pressure
TC: Total cholesterol
TG: Triglycerides
HDL-C: High-density lipoprotein cholesterol
LDL-C: Low-density lipoprotein cholesterol
Apo-B: Apolipoprotein B
MRI: Magnetic resonance imagingALB: albumin
GGT: Gamma-glutamyl transpeptidase
SCR: Serum creatinine
NAFLD: Non-alcoholic fatty liver disease
GAM: Generalised additive model
SMD: Standardized mean difference
OR: Odds ratio
CI: Confidence interval
AUC: Area under the curve
VAT: Visceral adipose tissue
SAT: Subcutaneous adipose tissue
PVAT: Perivascular adipose tissue
