# Supplementary material for: Association between metabolic score of visceral fat and carotid atherosclerosis in Chinese health screening population: a cross-sectional study
Source: BMC Public Health. 2024 Jun 28;24:1723. doi: 10.1186/s12889-024-19186-2 (PMC11212235; doi:10.1186/s12889-024-19186-2)
Supplement: Supplementary file 1 — Supplementary Material 1. [file 12889_2024_19186_MOESM1_ESM.zip › Additional Fig A1.pdf]

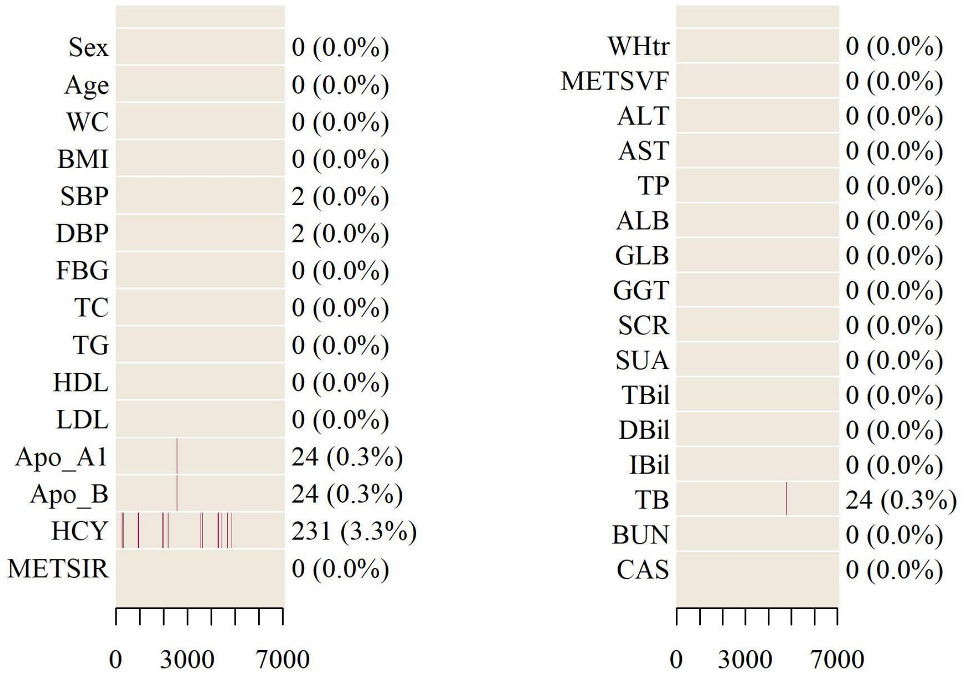

Administrator/2023-11-15 Administrator/2023-11-15

Fig A1.Distribution of missing data. WC, waist circumference; BMI, body mass index; SBP, systolic blood pressure; DBP, diastolic blood pressure; FBG, fasting blood glucose; TC, total cholesterol; TG, triglycerides; HDL-C, high-density lipoprotein cholesterol; LDL-C low-density lipoprotein cholesterol; Apo-A1, apolipoprotein-A1; Apo-B, apolipoprotein-B; HCY, homocysteine; METS-IR, metabolic score for insulin resistance; WHtr, waist-to-height ratio; ALT, alanine aminotransferase; AST, aspartate aminotransferase; TP, total protein; ALB, albumin; GLB, globulin; GGT, gamma-glutamyl transpeptidase; SCR, serum creatinine; SUA, seru uric acid; TBil, total bilirubin; DBil, direct bilirubin; IBil, indirect bilirubin; TB, total bile acids; BUN, blood urea nitrogen;CAS, carotid atherosclerosis.
