# Supplementary material for: Association between metabolic score of visceral fat and carotid atherosclerosis in Chinese health screening population: a cross-sectional study
Source: BMC Public Health. 2024 Jun 28;24:1723. doi: 10.1186/s12889-024-19186-2 (PMC11212235; doi:10.1186/s12889-024-19186-2)
Supplement: Supplementary file 2 — Supplementary Material 2. [file 12889_2024_19186_MOESM2_ESM.pdf]

Calculation example:

male, age:73, BMI:26.18kg/m2, FBG:98.1mg/dl, TG:169.1mg/dl, HDL-C:27.46mg/dl, WHtr:1.25

$$\mathrm{METS-IR} = \ln(2 * 98.1 + 169.1) * 26.18/\ln(27.46) = 46.67$$

$$\mathrm{METS-VF} = 4.466 + 0.011 * \ln(46.67)^3 + 3.239 * \ln(1.25)^3 + 0.319 + 0.594 * \ln(73) = 7.99$$
